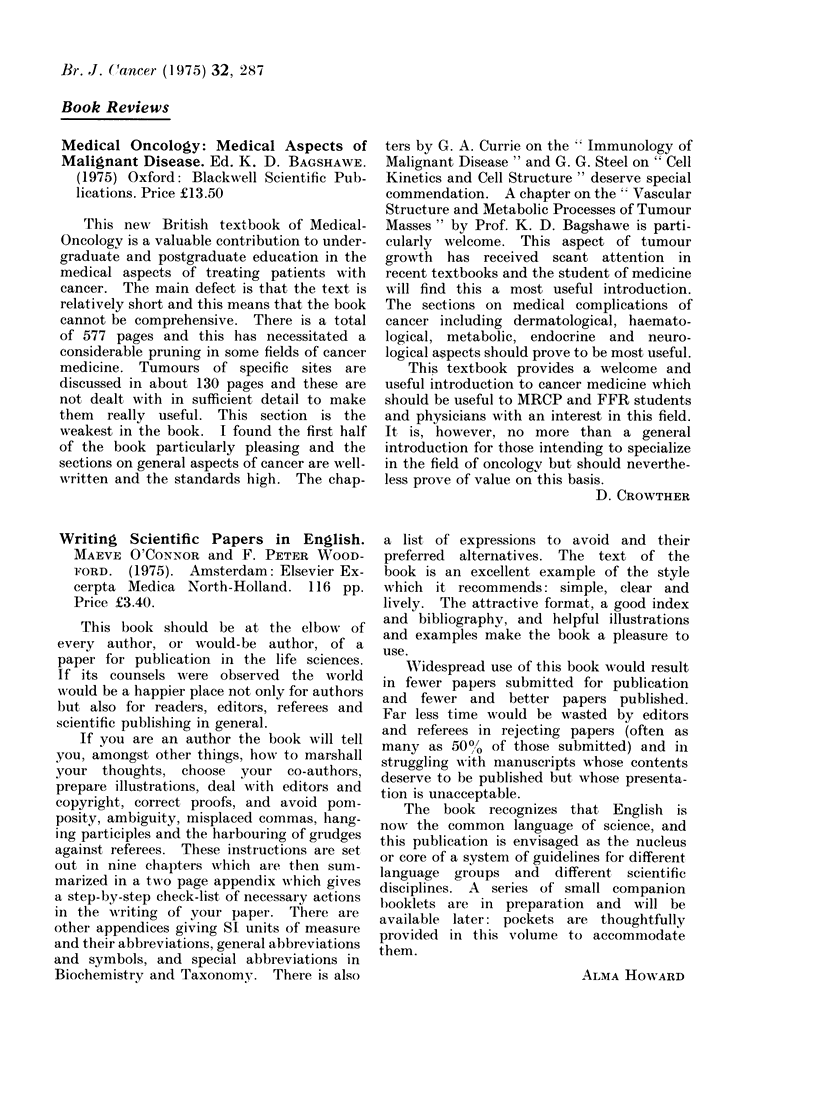# Medical Oncology: Medical Aspects of Malignant Disease

**Published:** 1975-08

**Authors:** D. Crowther


*Br. J. Cancer* (1975) **32**, 287

### *Book Reviews*

**Medical Oncology: Medical Aspects of Malignant Disease.** Ed. K. D. BAGSHAWE. (1975) Oxford: Blackwell Scientific Publications. Price £13.50

This new British textbook of Medical-Oncology is a valuable contribution to undergraduate and postgraduate education in the medical aspects of treating patients with cancer. The main defect is that the text is relatively short and this means that the book cannot be comprehensive. There is a total of 577 pages and this has necessitated a considerable pruning in some fields of cancer medicine. Tumours of specific sites are discussed in about 130 pages and these are not dealt with in sufficient detail to make them really useful. This section is the weakest in the book. I found the first half of the book particularly pleasing and the sections on general aspects of cancer are well-written and the standards high. The chapters by G. A. Currie on the " Immunology of Malignant Disease " and G. G. Steel on " Cell Kinetics and Cell Structure " deserve special commendation. A chapter on the " Vascular Structure and Metabolic Processes of Tumour Masses " by Prof. K. D. Bagshawe is particularly welcome. This aspect of tumour growth has received scant attention in recent textbooks and the student of medicine will find this a most useful introduction. The sections on medical complications of cancer including dermatological, haematological, metabolic, endocrine and neurological aspects should prove to be most useful.

This textbook provides a welcome and useful introduction to cancer medicine which should be useful to MRCP and FFR students and physicians with an interest in this field. It is, however, no more than a general introduction for those intending to specialize in the field of oncology but should nevertheless prove of value on this basis.

D. CROWTHER